# 229. Predictive markers for bacteremia in patients receiving extracorporeal membrane oxygenation (ECMO) support

**DOI:** 10.1093/ofid/ofad500.302

**Published:** 2023-11-27

**Authors:** Curtis Hendrix, Christopher Radcliffe, Hossam Tantawy, Scott C Roberts

**Affiliations:** Yale New Haven Hospital, New Haven, Connecticut; Massachusetts General Hospital, Boston, Massachusetts; Yale New Haven Hospital, New Haven, Connecticut; Yale School of Medicine, New Haven, Connecticut

## Abstract

**Background:**

Patients receiving extracorporeal membrane oxygenation (ECMO) support are critically ill, which can confound the usual metrics clinician use to diagnose bacteremia. The best methods to detect bacteremia in patients receiving ECMO support are unknown, and we sought to evaluate the accuracy of various metrics used in determining bacteremia.

**Methods:**

A retrospective review of patients receiving ECMO support at Yale New Haven Hospital from April 2020 to September 2021 who had blood cultures obtained for any reason was performed. Reasons for blood culture and metrics associated with bacteremia, including white blood cell count (WBC), maximum daily temperature, and procalcitonin, were evaluated for each blood culture event. If patients became bacteremic, only the first positive blood cultures were included in the analysis. Receiver operator curves for each metric were performed to generate areas under the curve (AUC) for assessing diagnostic accuracy.

**Results:**

60 patients who required ECMO support had 132 blood cultures performed, 22 (16.7%) with bacteremia and 110 (83.3%) without. In blood cultures with bacteremia compared to those without, there was no difference in WBC count (17.4 vs 17.9, p = 0.825), maximum daily temperature (37.1 vs 36.9 C, p = 0.572), or procalcitonin (2.0 vs 2.2, p = 0.776) on the day of culture. The most predictive reason for obtaining blood cultures with confirmed bacteremia was hemodynamic instability (AUC 0.668), followed by temperature dysregulation (AUC 0.623). Reasons for obtaining blood cultures minimally associated with bacteremia included adding ice to the ECMO circuit (AUC 0.527), leukocytosis (AUC 0.523), respiratory decompensation (AUC 0.500), surveillance culturing (AUC 0.432), and other reasons not specified but per clinician discretion (AUC 0.386).

**Conclusion:**

Hemodynamic instability as the reason for obtaining blood cultures was the most predictive of bacteremia in patients on ECMO support, while leukocytosis, temperature change, and surveillance cultures were less well associated. There was no difference in WBC counts, maximum daily temperature, and procalcitonin in patients with bacteremia versus those without. Optimizing clinical criteria to determine bacteremia in patients on ECMO remains challenging and warrants further study.

**Disclosures:**

**All Authors**: No reported disclosures

